# Anomalous Interlayer Exciton Diffusion in WS_2_/WSe_2_ Moiré Heterostructure

**DOI:** 10.1021/acsnano.4c00015

**Published:** 2024-07-01

**Authors:** Antonio Rossi, Jonas Zipfel, Indrajit Maity, Monica Lorenzon, Medha Dandu, Elyse Barré, Luca Francaviglia, Emma C. Regan, Zuocheng Zhang, Jacob H. Nie, Edward S. Barnard, Kenji Watanabe, Takashi Taniguchi, Eli Rotenberg, Feng Wang, Johannes Lischner, Archana Raja, Alexander Weber-Bargioni

**Affiliations:** † The Molecular Foundry, Lawrence Berkeley National Laboratory, Berkeley, California 94720, United States; ‡ Advanced Light Source, Lawrence Berkeley National Laboratory, Berkeley, California 94720, United States; § Center for Nanotechnology Innovation @ NEST, Instituto Italiano di Tecnologia, 56127 Pisa, Italy; ∥ Imperial College London, South Kensington Campus, London SW7 2AZ, U.K.; ⊥ Department of Physics, University of California at Berkeley, Berkeley, California 94720, United States; # Department of Physics, University of California at Santa Barbara, Santa Barbara, California 93106, United States; ∇ Research Center for Functional Materials, National Institute for Materials Science, 1-1 Namiki, Tsukuba 305-0047, Japan; ○ International Center for Materials Nanoarchitectonics, National Institute for Materials Science, 1-1 Namiki, Tsukuba 305-0047, Japan

**Keywords:** van der Waals heterostructures, exciton diffusion, phasons, moiré potential, photoluminescence, interlayer exciton

## Abstract

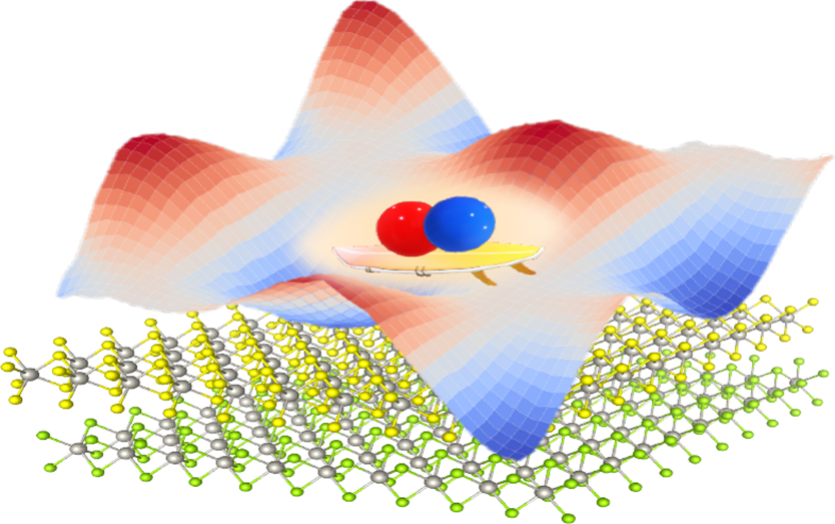

Stacking van der Waals crystals allows for the on-demand creation of a periodic potential landscape to tailor the transport of quasiparticle excitations. We investigate the diffusion of photoexcited electron–hole pairs, or excitons, at the interface of WS_2_/WSe_2_ van der Waals heterostructure over a wide range of temperatures. We observe the appearance of distinct interlayer excitons for parallel and antiparallel stacking and track their diffusion through spatially and temporally resolved photoluminescence spectroscopy from 30 to 250 K. While the measured exciton diffusivity decreases with temperature, it surprisingly plateaus below 90 K. Our observations cannot be explained by classical models like hopping in the moiré potential. A combination of ab initio theory and molecular dynamics simulations suggests that low-energy phonons arising from the mismatched lattices of moiré heterostructures, also known as phasons, play a key role in describing and understanding this anomalous behavior of exciton diffusion. Our observations indicate that the moiré potential landscape is dynamic down to very low temperatures and that the phason modes can enable efficient transport of energy in the form of excitons.

Van der Waals (vdW) heterostructures are often considered key to unlock the full potential of two-dimensional (2D) materials.^1,2^ Weak interaction at the interface makes it possible to virtually stack any 2D system on top of another, creating artificial heterostructures with entirely novel properties. Twisted bilayer graphene (TBG), for example, displays flat bands near the Fermi level.^3^ The heavy Fermions populating these states not only give rise to unconventional superconductivity^4^ but also insulating behavior driven by electron correlations.^5^ Strong electron correlation is also observed in transition metal dichalcogenide (TMD) heterostructures like WS_2_/WSe_2_, where flat bands arise^6,7^ and a Mott insulator state and generalized Wigner crystallization are observed.^8,9^ These and many other emergent properties,^10−12^ typically unexpected in the constituent monolayers, have been attributed to the moiré potential created by the superposition of two layers with different lattice constants and/or relative crystal orientation.^13,14^ In this paper, we show that the moiré potential is not stationary, and its dynamical nature is inferred by employing the temperature-dependent propagation of interlayer excitons (IX) in WS_2_/WSe_2_ as the probe for the moiré potential dynamics. Our experiments suggest that the moiré potential is dynamic at finite temperature and the quasiparticle describing its motion, the phason,^15,16^ strongly affects the exciton diffusion mechanism at low temperatures.

The WS_2_/WSe_2_ system is made up of stacked direct band gap monolayers and can host both intralayer excitons and IX.^17,18^ The latter is formed when the electron and hole reside in different layers. The IX’s lifetime is extremely long^19,20^ compared to the few ps measured for monolayer TMDs^21^ and can reach hundreds of nanoseconds in some systems.^22^ Its spectroscopic and dynamical features are dramatically affected by the presence of a moiré potential, as opposed to the exciton diffusion in a single monolayer where such moiré modulation is not present.^23,24^ An initial study via transient absorption spectroscopy of IX dynamics and diffusion in CVD-grown WS_2_/WSe_2_ suggested that the moiré potential can trap excitons inhibiting their diffusion.^25^ Furthermore, a trapping/detrapping process, which is a temperature-activated mechanism where thermal energy allows the exciton to hop across the moiré potential wells, has been proposed for a MoSe_2_/WSe_2_ heterostructure at various twist angles.^26^ Our observations of IX diffusion cannot be explained by such a model alone. For that reason, we consider the role of phonons, specifically of moiré-induced phonons also known as phasons,^15^ that are still experimentally unexplored. Recent calculations demonstrate how these quasiparticles can play a crucial role in understanding the charge ordering and correlated states of magic-angle TBG.^27,28^

We perform a wide-ranging temperature-dependent study of the IX diffusion as observed through photoluminescence (PL). We explore the PL spectra of the interlayer excitons as a function of temperature and stacking angle (0° and 60°), together with the lifetime and diffusion length from which the diffusion coefficient is derived.^29^ This allows us to gain insight, first, into the temperature-dependent spectral properties of the IX for the two stacking configurations with different moiré landscape and then into the IX propagation dynamics. Our findings go beyond the classical Arrhenius model of thermally activated exciton transport involving a simple fixed potential landscape that have been reported in previous works where exciton diffusion is negligible at low temperatures (below 100 K).^25,26,30^ Instead, we find that below 100 K excitons can explore the system, despite being fully trapped inside deep moiré potentials. An accurate model describing the moiré potential landscape is also provided, matching the energy barrier obtained from temperature-dependent diffusion measurements.

Finally, a mechanism that describes the electronic properties and vibrational modes of the two systems is proposed, showing how the moiré potential itself is affected by temperature and is likely the main mechanism for a nonzero diffusion coefficient at cryogenic temperatures, where the excitons are fully trapped.

## Results & Discussion

**Figure 1:**
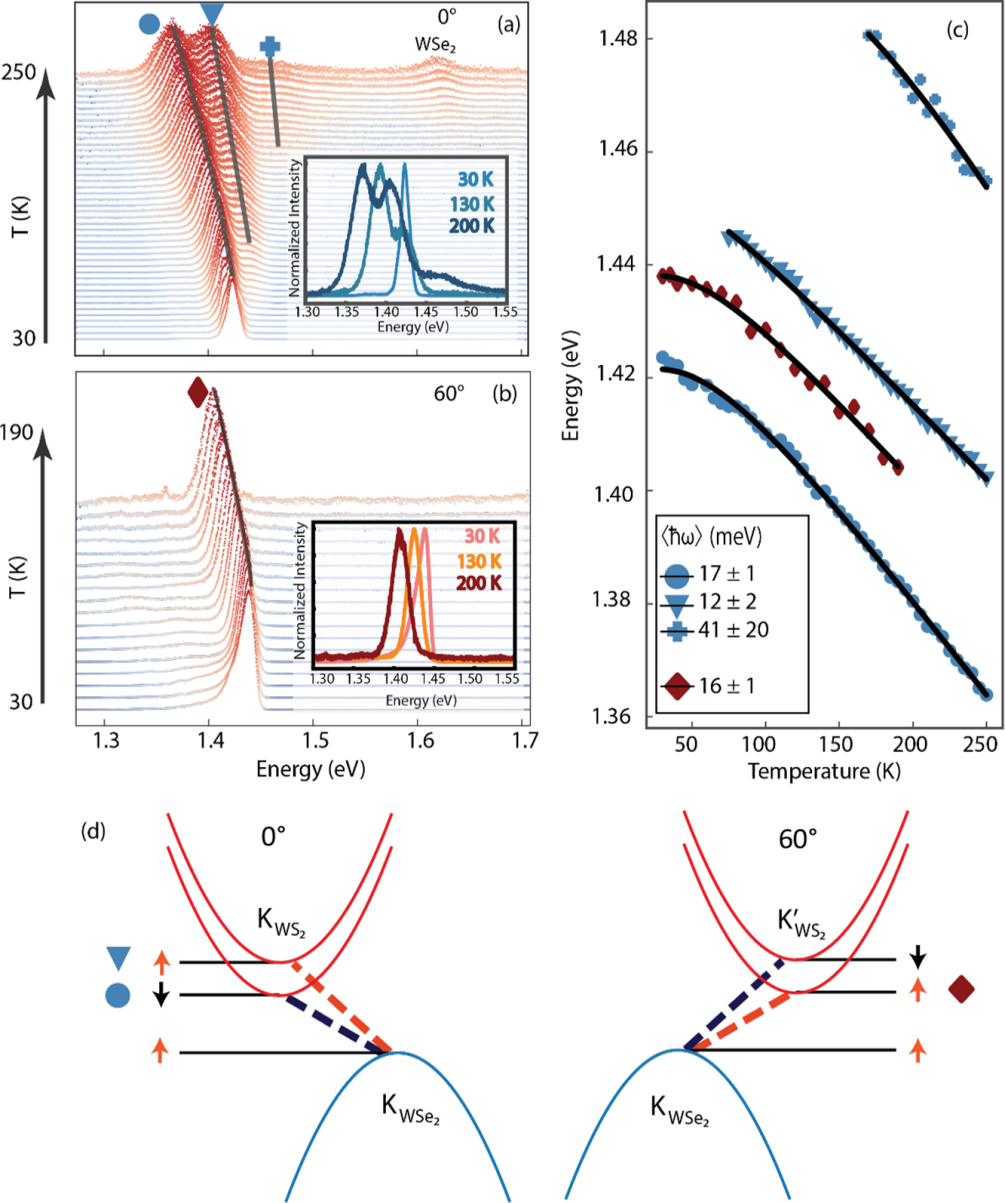
PL spectra of 0° (a) and 60° (b) stacking angles at different temperatures. Insets display representative spectra collected at different temperatures. Blue and red symbols identify the different peaks present in the spectra. In panel (a), a WSe_2_ intralayer exciton peak is highlighted at higher temperatures. (c) Peak positions tracked as a function of temperature, fitted with the Varshni equation (black line). Inset displays the value of the average phonon energy for each fit. (d) Scheme of the spin-polarized band structure for the 0° and 60° (left and right panels, respectively) structures with the corresponding vertical optical transitions (dashed lines).

The IX is an electron–hole pair where the two charged particles are in different layers, and the recombination is affected by the selection rules dictated by the twist angle. In fact, the different twist angles affect the electronic properties of the heterostructure, dictating the alignments of the spin-valley locked bands. To visualize the IX spectral properties, we analyze the PL emission as a function of temperature. Figure 1a,b reports the PL spectra of the two WS_2_/WSe_2_ heterostructures with 0° and the 60° alignment in blue and orange, respectively (Figure S1). The PL spectra are reported at increasing temperatures, starting from 30 K. For both stackings, the emission is entirely dominated by the IX involving the top of the WSe_2_ valence band (hole) and the bottom of the WS_2_ conduction band (electron) at K and K’. Such emission is centered at 1.42 eV for the 0° stacking and 1.44 eV for the 60° stacking at 30 K, and progressively, red shifts with increasing temperature, as expected.^31,32^ It is important to note that the 0° and 60° stacking configurations exhibit different dielectric screening due to the distinct electronic environments that they create. Consequently, there is a variation in their exciton binding energies,^33,34^ highlighting the need for careful comparison between these structures. Moreover, the exfoliation and stacking method employed in device fabrication also introduces variability in coupling strength between layers , potentially leading to observable differences in emission energy. Interestingly, for the 0° structure, two thermally activated IXs emerge: a first one at around 75 K and a second one above 150 K. The insets of Figure 1a,b show representative spectra featuring all peaks at three selected temperatures. We can describe the peak evolution by tracking their position as a function of temperature, as displayed in Figure 1c. We evaluate each peak position by fitting the spectra with one, two, or three Voigt functions, depending on the number of observed peaks (Figure S2). Using Varshni’s equation, which empirically describes the temperature dependence of energy gaps in semiconductors,^32^ we extract an empirical electron–phonon coupling coefficient associated with the average phonon energy ⟨*ℏ*ω⟩. The fitted parameter values are reported in the inset of panel (c). The two lowest excitons from the 0° structure (marked with blue circles and triangles in Figure 1a) have similar fitting parameters, suggesting very similar electron–phonon coupling. Moreover, they display an energy separation of Δ*E* ∼ 30 meV, compatible with the spin orbit splitting of the conduction band in WS_2._^35^ The origin of the third, highest energy peak marked by a blue cross, which displays a much steeper slope, is unclear as of now; however, it could be associated with one of the moiré excitons previously observed in the same kind of heterostructure for the intralayer exciton.^11^ Another plausible hypothesis considers the description of the moiré potential as a quantum well; within this hypothesis, the third peak arises from the optical transition involving the first excited state of the well, similarly to what reported by Chatterjee and co-workers.^36^ The PL spectra collected for the 60° stacking sample show different behavior. There is only one peak (marked with red diamond), and it shows an identical trend as the two lowest energy emission peaks from the 0°sample. The presence of three peaks in the 0° alignment and the single peak emission for the 60° can be ascribed to the selection rules coming from the electronic structure of the single layers.^37,38^ The lowest energetic transition for the 0° sample should be related to the spin-dark exciton. However, the presence of the moiré potential has been demonstrated to partially lift those selection rules in systems with large lattice mismatch,^37−40^ and given it is the lowest energy state, its high population at low temperatures makes the PL emission comparably strong, similarly to what observed for MoSe_2_/WSe_2_ structure.^41^ With increasing temperature, however, the tail of the Fermi–Dirac distribution starts populating the higher spin-split level of the WS_2_ conduction band, allowing the spin-aligned IXs to become observable.^18^ On the other hand, the 60° sample has a bright transition as the lowest available one, allowing all the hot electrons to relax and recombine through that channel, while the higher spin-dark state, being much less efficient and much less populated even for elevated temperatures, is not easily observed (Figure 1d). It is crucial to note that the extended lifetime of excitons, as discussed later in the paper, further facilitates the relaxation from the spin-bright to the spin-dark state in the 0° sample. This longevity allows excitons more time to undergo this transition, augmenting the population of the spin-dark state and influencing the observed PL emission patterns.

We now focus our attention on IX dynamics, studying its diffusion and recombination lifetime. We perform an exciton diffusion study by collecting the light associated with the IX transitions only and mapping it in real space (Figures S3 and S4). We use a 790 nm long pass filter in detection to cut out any potential light emitted from the intralayer transitions, particularly at elevated temperatures.

**Figure 2:**
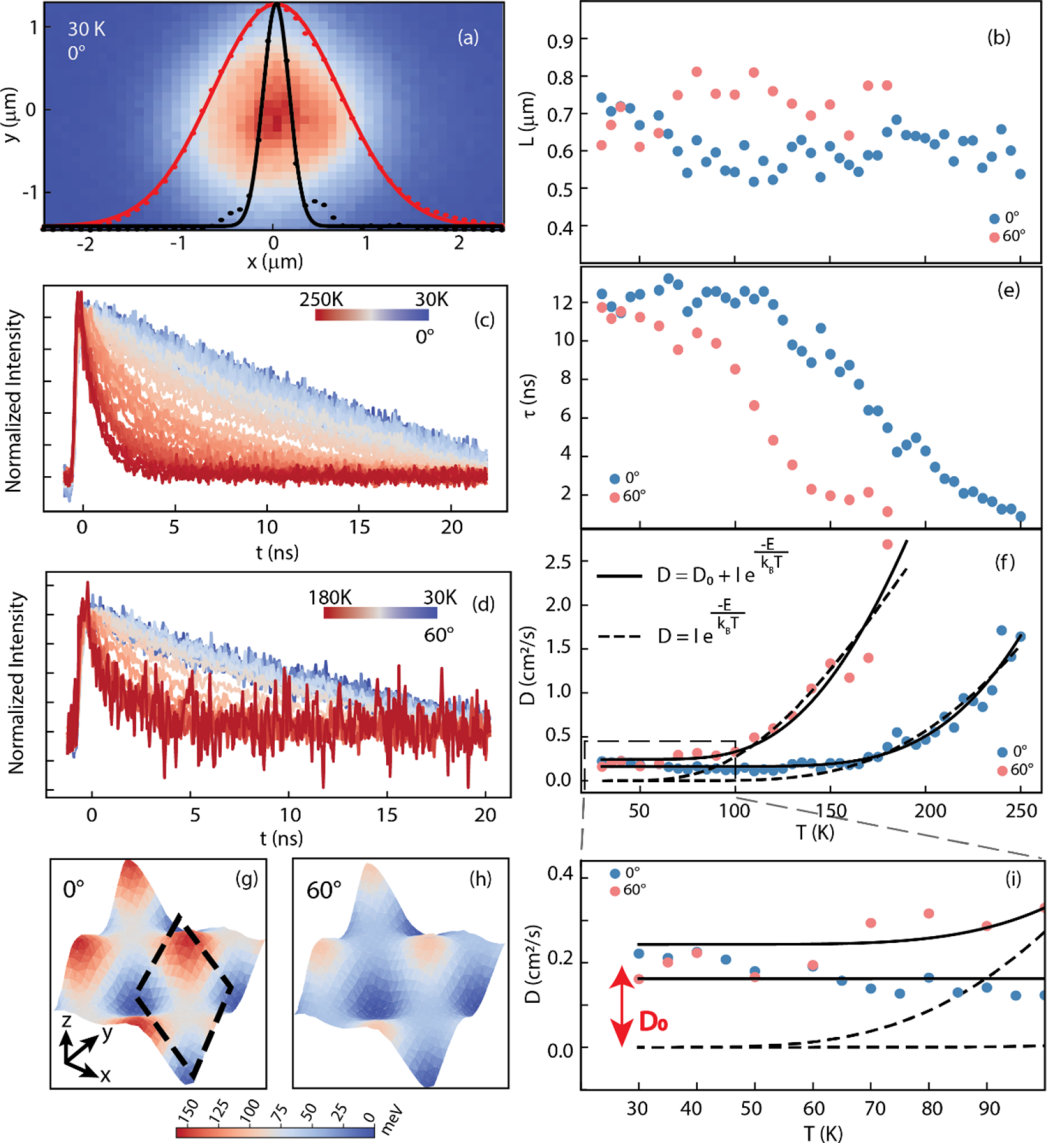
(a) Exciton diffusion length evaluated from PL area with respect to the laser excitation width at 30K. (b) Diffusion length values at different temperatures for the two stacking angles. PL lifetime traces at different temperatures for 0° (c) and 60° (d) stacking angle. (e) PL recombination lifetime as a function of temperature for the two stacking angles. (f) Diffusivity values fitted with (solid line) and without (dashed line) *D*_0_ offset. (g) and (h) moiré potential evaluated as described in the main text for 0° and 60°, respectively. Dashed black diamond indicates the moiré unit cell of periodicity λ ∼ 7 nm. (i) Zoom-in of the diffusivity map at a lower temperature range to highlight the *D*_0_.

The diffusion length is defined as^42^
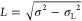
1where σ_L_ is the Gaussian covariance that fits the centrosymmetric excitation laser profile along an arbitrary axis (Figure 2a) and σ is the Gaussian covariance of the real space PL intensity, evaluated in the same manner, assuming that, if excitons can diffuse, the emission of the initially excited 2D Gaussian profile broadens isotropically over time, following Fick’s second law. The diffusion length *L* is evaluated as the square root of the difference between the squared covariances, according to eq 1 (Figure 2b).

Simultaneously, we collected the time-resolved PL emission. Figure 2c,d shows data for the 0°and 60° samples, respectively. The decay traces exhibit a single exponential trend, which we fit to extract PL lifetime τ (Figure 2e).

The diffusion length for both stacking angles is largely unaffected by temperature variations. It is mostly confined within a mean value of ∼600 nm, which is consistent with reported diffusion lengths in monolayer systems.^43,44^ On the other hand, the PL lifetime shows a clear decreasing trend with increasing temperature, dropping after 150 K for 0° and 100 K for 60°. Using these values of *L* and τ, we extract the diffusion coefficient, defined as^43,45^
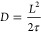
2

Figure 2f shows the calculated values of diffusion coefficient D (diffusivity), which undergoes a thermally activated process resulting in a pronounced increase of the diffusivity with increasing temperature. Such an exponential behavior is expected when considering a simple moiré-trap picture.^25,26,30^ Once formed, the IXs are affected by the presence of the moiré potential landscape and are trapped in its local minima. However, if enough thermal energy is provided and the activation barrier is overcome, the IX can diffuse faster, reaching a value comparable with the one of intralayer excitons of an isolated monolayer where the moiré potential is not present (Figure S5). To evaluate the effective potential for diffusion, we perform extensive density functional theory (DFT) calculations for both 0° and 60° twisted heterobilayers, considering the atomic reconstructions at *T* = 0 (see the Supporting Information). The moiré potential is evaluated as
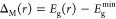
3

*E*_g_(*r*) represents the local bandgap and *E*_g_^min^ represents the averaged bandgap.^37^ Since Δ_M_(*r*) varies smoothly over the moiré period, the effective moiré potential for diffusion can be extracted from the local bandgap at different high-symmetry stackings. The averaged effective moiré potential for 0° (124 meV) is unmistakably larger than that of 60° WS_2_/WSe_2_ (85 meV) (Figure 2g,h).

The most striking observation, however, is that a simple detrapping model is not consistent with what observed at low temperatures (*T* < 100 K). Figure 2i displays the diffusivity data reported in the dashed box in Figure 2f. We emphasize that an Arrhenius-like description would make *D* tend to 0 at low temperatures (dashed line). However, the data clearly show a finite offset *D*_0_, indicating that the IXs are diffusing, even at low temperatures. The diffusivity as a function of temperature now takes the form:
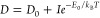
4

The moiré potential height *E*_0_ can therefore be extracted from the data resulting in a value of 126 and 67 meV for 0° and 60° orientation, respectively, very similar to the theoretically calculated potential reported above and shown in Figure 2g,h. Values of *D*_0_ = 0.16 and 0.24 cm^2^/s are also obtained from the fitting for 0° and 60°, respectively. To understand the nature of *D*_0_, we aim to juxtapose our findings against existing models detailed in the literature. Recent investigations into TMDs have highlighted the significance of finite diffusion coefficients, with substantial focus directed toward monolayer systems.^46−48^ These studies have contributed to understanding exciton transport mechanisms within these materials, including effects such as weak localization. Our work extends this exploration to heterobilayer TMDs, a domain where the moiré potential, not present in monolayer systems, notably influences IX behavior. In our heterobilayer system, exciton transport at high temperatures is characterized by thermally activated hopping across moiré potential minima, demonstrating an Arrhenius-type temperature dependence. This established mechanism, however, does not account for the observed finite diffusivity at lower temperatures. Unlike monolayers where phonon scattering could facilitate exciton transport, the heterobilayer’s thermal phonon energy scales fall significantly below the moiré barriers evaluated above, suggesting an alternative transport pathway. Other effects such as impurities, defects, and disorder play a less critical role in our heterobilayer observations. This conclusion is supported by other analyses showing that these elements would typically lead to exciton trapping^49^ and reduced diffusivity at low temperatures contrary to our findings of maintained finite diffusivity, suggesting minimal trapping within our system. Furthermore, our examination of quantum effects, supported by low exciton density estimations, indicates the absence of significant exciton–exciton interactions. In fact, we estimate the exciton density (as reported in the Experimental Methods section) to be significantly low (5 × 10^10^ cm^–2^), much smaller than one exciton per moiré unit cell. This assessment is corroborated by the photoluminescence spectrum of the WSe_2_ monolayer, which lacks any signs of exciton–exciton peaks^50^ under experimental conditions similar to those of our IX studies (see Figure S6). Other effects such as exciton–electron interaction that have been demonstrated to lead to an increase in the diffusion coefficient with increasing carrier density^48^ can also be excluded. In fact, the PL spectrum of monolayer WSe_2_ displayed in Figures S5 and S6 does not display the typical feature of the charged exciton peak arising from the presence of high free carrier density.^51^

We propose moiré phonons (phasons)^15^ to be responsible for the deviation from the Arrhenius description. The phason modes represent an effective translation of the moiré sites due to the nonuniform out-of-phase in-plane translation of two constituent layers in the heterobilayer. A mechanism described as “moiré surfing” has been proposed to affect electron transport in twisted TMD homobilayer.^52^ It is unique to moiré systems, where thermally populated phason modes enable the collective motion of the moiré landscape, thereby, in our case, transporting excitons. This process diverges fundamentally from the scattering phenomena previously considered for monolayers, offering another perspective on exciton mobility in complex TMD systems.

**Figure 3:**
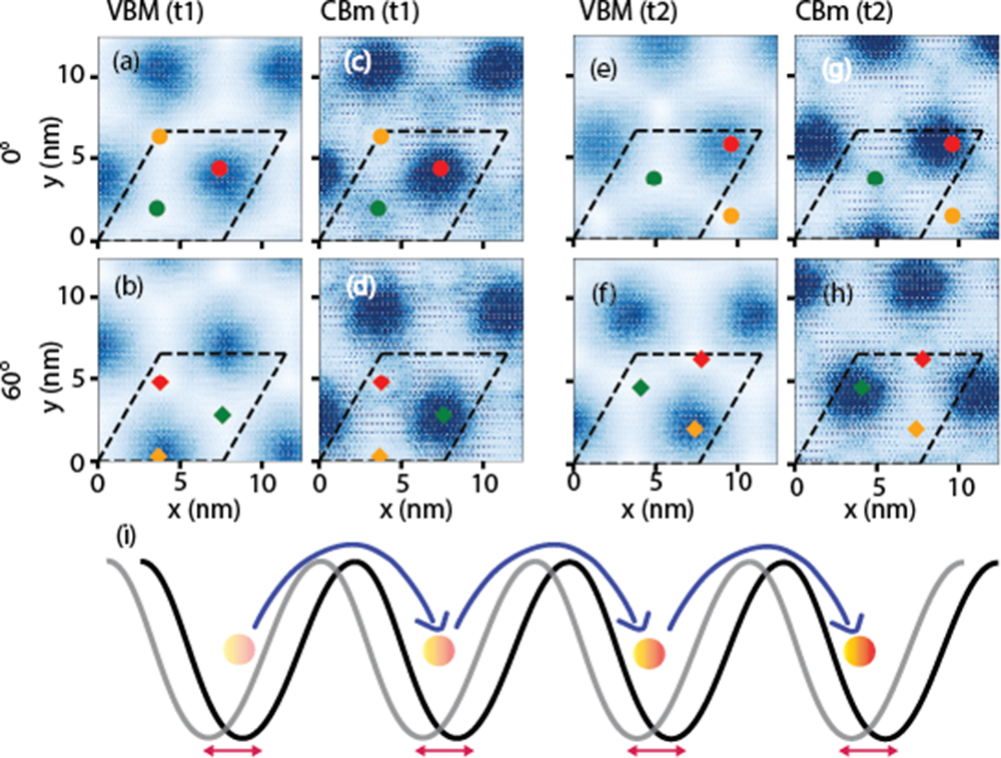
Local holes and electron densities evaluated at valence band maxima (VBM) and conduction band minima (CBM), respectively, for 0° and 60° deg structure. VBM and CBM densities are evaluated at different times, *t*_1_ (a–d) and *t*_2_ (e–h) for finite temperature *T* = 175 K with *t*_2_ – *t*_1_ = 0.3 ns. Colored dots and diamonds track the different moiré sites of the heterostructure. (i) Scheme of the exciton (yellow sphere) transport (blue arrows) assisted by moiré phasons (red arrows).

Here, we calculate that the phason modes give rise to dynamic moiré potentials at finite temperature. We simulate a WS_2_/WSe_2_ heterostructure in both twist angle configurations in a canonical ensemble using classical molecular dynamics (MD) simulations. We find that the moiré pattern starts to move in-plane due to the time-dependent relative local displacement of the two layers, as a result of the thermally activated phasons. Figure 3a–h shows different temporal snapshots of the valence band maximum (VBM) and conduction band minimum (CBM) single particle density of states for 0° and 60° stacking at a lattice temperature of 175 K. The MD simulation reveals, as evidenced by the colored marks, that the different stacking sites, and thus the moiré potential, move with time, affecting the exciton hopping (Figure 3i). It is also shown that the single particle density of states calculated from DFT strictly follows this movement as would be expected within the Born–Oppenheimer approximation (see Supporting Information Figure S9. An animated simulation is found in the online Supporting Information representing the crystal dynamics over 2 ns at 175 K).

**Figure 4:**
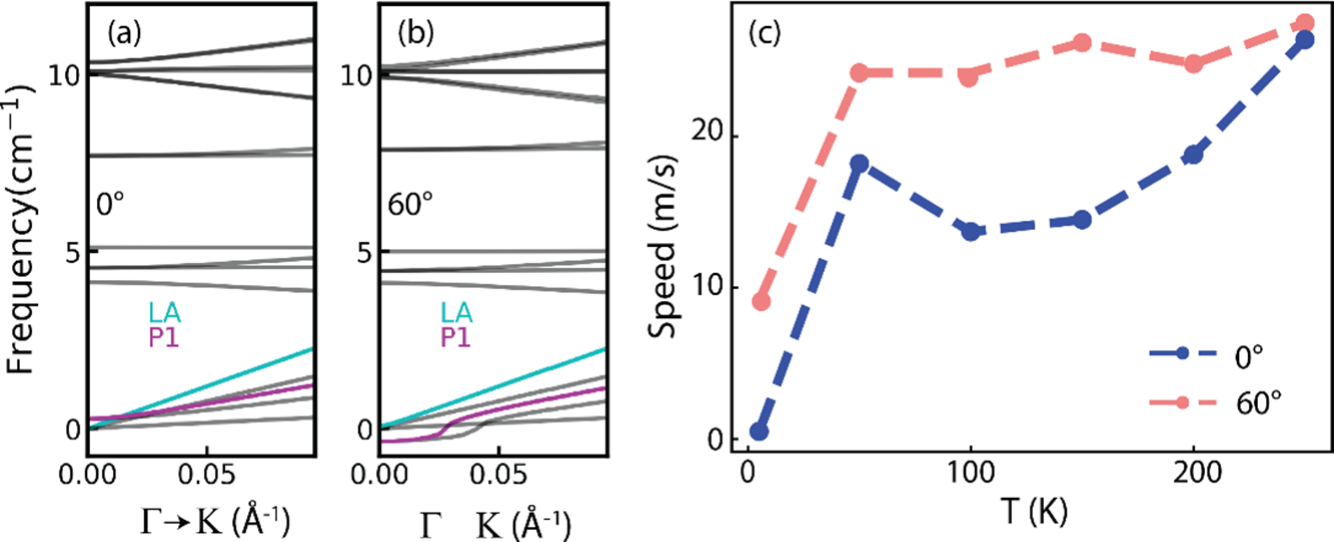
(a,b) Phason dispersion overlaid to the phonon dispersion for 0° and 60°, respectively, computed at *T* = 0 K for the long-wavelength phonons near the Γ point. Phason speed is reduced by a factor of 2 and 1.6 when compared to the LA mode for 0° and 60° twist angles, respectively. (c) Speed of moiré sites captured using molecular dynamics simulations as a function of temperature for 0° and 60°.

As a preliminary indication of our model’s accuracy, we compare the speed of phasons derived from phason dispersion calculations (Figure 4a,b) and MD simulations with the experimentally observed value. By calculating the phason group velocity directly from the phonon dispersion curve (Figure S10), we estimate the speed to be up to 200 m/s. Note that the phason has a parabolic dispersion close to the Γ point of the BZ corresponding to a vanishing group velocity reflecting the small, but finite energy to slide a commensurate moiré bilayer. It is also possible to extract the speed of the moiré phason as a function of temperature from the MD simulation by evaluating the stacking site displacement at different times (Figure 4c). The speed increases sharply with temperature and then quickly saturates, in agreement with the expectation that the sliding of two dissimilar layers costs little energy, which is generally referred to as super lubricant behavior.^53,54^ The value obtained from the MD simulations is considerably lower, with respect to the fitting of the phason acoustic branch, approximately 20 m/s. The reason behind this mismatch is rooted in the parabolic dispersion around Γ, deviating from a typical linear phonon acoustic branch, whose slope is the speed of sound. It is notable that the speed estimated from the experimental measurements—obtained by dividing the diffusion length by the radiative lifetime—is found to be around 50 m/s, which lies within the bounds set by the two theoretical methodologies described above. The agreement of our experimental results with the boundaries defined by our simulations underscores the necessity of careful handling and interpretation of experimental results when dealing with moiré systems.

## Conclusions

This paper studies the IX diffusion in WS_2_/WSe_2_ heterostructures at two different stacking angles as a function of temperature. The distinct PL signatures we have observed are tied to the intrinsic selection rules governing the monolayers that constitute these heterostructures. Intriguingly, we have determined that the moiré potential appears to exhibit different depths for the two stacking angles. Specifically, a shallower potential of 67 meV is associated with the 60° stacking, while a deeper 126 meV potential characterizes the 0° stacking. Unexpectedly, the most striking observation is the existence of an anomalous nonzero diffusion at cryogenic temperatures in samples of both orientations. This cannot be explained within the conventional framework of an activated process that detraps the excitons from the moiré potential well. To make sense of this unconventional behavior, we suggest that IX may be effectively transported across the crystal lattice by a moving moiré potential landscape. Our MD simulations indicate that this process may initiate from temperatures as low as 5 K. Lower temperature studies of IX dynamics may further elucidate the complex relationship between excitons and the phonons of moiré systems. The consistency we have observed between theory and experiment, considering the matching moiré potential barrier and moiré speed, serves as a compelling indication that moiré phonons, phasons, are responsible for the anomalous diffusion observed in this work. These findings underscore the critical significance of incorporating phason dynamics into forthcoming investigations of moiré systems.

## Experimental Methods

The heterostructure is prepared with a standard pick up technique.^55^ The TMD heterostructure is incapsulated in h-BN to preserve the pristine properties of the system. The relative alignment has been checked using second harmonic generation (Figure S1). The experiment has been carried out using a Montana Cryostation s200 closed-cycle helium cryostat with full temperature control down to 4.2 K and 100x optical magnification (NA = 0.95). The samples are excited using a pulsed (38.9 MHz) supercontinuum laser tuned to 510 nm with 10 nm line width and 30 ps pulse length, focused down to a spot diameter of about 320 nm fwhm, yielding an energy density per pulse of 0.38 μJ/cm^2^ for our diffusion experiment. Assuming 5% absorption mainly from the WS_2_ B-exciton and unity conversion into interlayer excitons, this amounts to an exciton density of roughly 5 × 10^10^ cm^–2^, within a regime where we can exclude a nonlinear response. At this density, quantum effects due to dipole–dipole interaction are negligibly small compared to the thermal energy in the range of temperatures explored.^56^ PL spectra are collected using a spectrometer with a dispersion grating together with a Peltier-cooled charge-coupled device (CCD) camera, while lifetimes are measured using an avalanche photo diode (APD) with a nominal temporal resolution of 30 ps. Real space imaging of the PL emission to extract exciton diffusion lengths is realized using a fast and highly efficient CMOS detector in the time integrated detection mode.

## Computational Methods

Twisted WS_2_/WSe_2_ heterobilayers have been generated using the TWISTER package.^57^ All of the structural relaxations, molecular dynamics, and phonon calculations are performed using classical interatomic potentials fitted to DFT calculations. The intralayer interactions within WSe_2_ and WS_2_ are described using Stillinger-Weber potential,^58^ and the interlayer interactions are captured using the Kolmogorov-Crespi potential.^58^ All the simulations with classical interatomic potentials are performed using the LAMMPS package,^59^ and the phonon calculations are performed using a modified version of the PHONOPY package.^60^ We relax the atoms within a fixed simulation box with the force tolerance of 10^–6^ eV/Å for any atom along any direction. All the MD simulations are performed in the canonical ensembles for several temperatures, and the moiré movements are extracted in the microcanonical ensemble. The speed of moiré sites is extracted from MD simulations of a 3 × 3 × 1 supercell of the moiré unit cell. We find that the moiré sites move even for a simulation of a 20 × 20 × 1 supercell. Note that the supercell size dictates the lowest accessible momentum close to Γ point, i.e., *q*_min_ ∝ 1 *L*_m_, where *L*_m_ is the moiré simulation cell lattice constant. The moiré site speed differences between twist angles 0° and 60° are reproduced for 1 × 1 × 1 and 6 × 6 x 1 supercells as well. All the electronic structure calculations are performed using the SIESTA package.^61^ All the calculations include spin–orbit coupling.^62^ We have used the local density approximation with the Perdew–Zunger parametrization as the exchange-correlation functional.^63^

## Data Availability

The twisted heterobilayer structure construction, atomic relaxations, molecular dynamics simulations, phonon spectra calculations, and electronic band structure calculations presented in the paper were carried out using publicly available codes. Our findings can be fully reproduced using these codes. Some of the postprocessing tools, such as tracking the motion of moiré sites, developed for the paper will be made publicly available at https://gitlab.com/_imaity_/Moiredynamics.

## Supporting Information Available

Device characterization measurements to determine the layers alignment, PL fitting scheme, exciton diffusion measurements for each temperature, PL spectra of monolayer WSe_2_ at different excitation power, and computational details for the determination of moiré potential and exitonic band structure (PDF)
Displaying 2 ns moiré dynamics at finite temperature (MP4)

## Notes

An earlier version of this work was submitted and is available on preprint servers. For further reference, see: A.R.; J.Z.; I.M.; M.L.; L.F.; E.C.R.; Z.Z.; J.H.N.; E.B.; K.W.; T.T.; E.R.; F.W.; J.L.; A.R.; A.W.-B., phason-mediated interlayer exciton diffusion in WS_2_/WSe_2_ moiré heterostructure. 2023, Article Number 2301.07750. arXiv. https://arxiv.org/abs/2301.07750 (accessed May 9, 2024). A.R.; J.Z.; I.M.; M.L.; L.F.; E.C.R.; Z.Z.; J.H.N.; E.B.; K.W.; T.T.; E.R.; F.W.; J.L.; A.R.; A.W.-B., Anomalous interlayer exciton diffusion in WS_2_/WSe_2_ moiré heterostructure. 2023, Article Number rs-2627775/v1. Research Square. https://www.researchsquare.com/article/rs-2627775/v1 (accessed May 9, 2024).

## References

[ref1] Novoselov K. S.; Mishchenko A.; Carvalho A.; Castro Neto A. H. 2D materials and van der Waals heterostructures. Science 2016, 353, aac9439 10.1126/science.aac9439. 27471306

[ref2] Liu Y.; et al. Van der Waals heterostructures and devices. Nat. Rev. Mater. 2016, 1, 16042 10.1038/natrevmats.2016.42.

[ref3] Bistritzer R.; MacDonald A. H. Moiré bands in twisted double-layer graphene. Proc. Natl. Acad. Sci. U.S.A. 2011, 108, 12233–12237. 10.1073/pnas.1108174108. 21730173 PMC3145708

[ref4] Cao Y.; et al. Unconventional superconductivity in magic-angle graphene superlattices. Nature 2018, 556, 43–50. 10.1038/nature26160. 29512651

[ref5] Codecido E.; et al. Correlated insulating and superconducting states in twisted bilayer graphene below the magic angle. Sci. Adv. 2019, 5, eaaw9770 10.1126/sciadv.aaw9770. 31799399 PMC6868676

[ref6] Miao S.; et al. Strong interaction between interlayer excitons and correlated electrons in WSe2/WS2 moiré superlattice. Nat. Commun. 2021, 12, 3608 10.1038/s41467-021-23732-6. 34127668 PMC8203657

[ref7] Stansbury C. H.; et al. Visualizing electron localization of WS2/WSe2 moiré superlattices in momentum space. Sci. Adv. 2021, 7, eabf4387 10.1126/sciadv.abf4387. 34516763 PMC8442863

[ref8] Regan E. C.; et al. Mott and generalized Wigner crystal states in WSe2/WS2 moiré superlattices. Nature 2020, 579, 359–363. 10.1038/s41586-020-2092-4. 32188951

[ref9] Tang Y.; et al. Evidence of frustrated magnetic interactions in a Wigner–Mott insulator. Nat. Nanotechnol. 2023, 18, 233–237. 10.1038/s41565-022-01309-8. 36646827

[ref10] Tang Y.; et al. Simulation of Hubbard model physics in WSe2/WS2 moiré superlattices. Nature 2020, 579, 353–358. 10.1038/s41586-020-2085-3. 32188950

[ref11] Naik M. H.; et al. Intralayer charge-transfer moiré excitons in van der Waals superlattices. Nature 2022, 609, 52–57. 10.1038/s41586-022-04991-9. 36045239

[ref12] Debnath R.; et al. Tuning exciton complexes in twisted bilayer WSe_2_ at intermediate misorientation. Phys. Rev. B 2022, 106, 125409 10.1103/PhysRevB.106.125409.

[ref13] Xiao Y.; Liu J.; Fu L. Moiré is More: Access to New Properties of Two-Dimensional Layered Materials. Matter 2020, 3, 1142–1161. 10.1016/j.matt.2020.07.001.

[ref14] Shabani S.; et al. Deep moiré potentials in twisted transition metal dichalcogenide bilayers. Nat. Phys. 2021, 17, 720–725. 10.1038/s41567-021-01174-7.

[ref15] Maity I.; Naik M. H.; Maiti P. K.; Krishnamurthy H. R.; Jain M. Phonons in twisted transition-metal dichalcogenide bilayers: Ultrasoft phasons and a transition from a superlubric to a pinned phase. Phys. Rev. Research 2020, 2, 013335 10.1103/PhysRevResearch.2.013335.

[ref16] Ochoa H. Moiré-pattern fluctuations and electron-phason coupling in twisted bilayer graphene. Phys. Rev. B 2019, 100, 155426 10.1103/PhysRevB.100.155426.

[ref17] Jin C.; et al. Observation of moiré excitons in WSe2/WS2 heterostructure superlattices. Nature 2019, 567, 76–80. 10.1038/s41586-019-0976-y. 30804525

[ref18] Yu J.; et al. Observation of double indirect interlayer exciton in WSe_2_/WS_2_ heterostructure. Opt. Express, OE 2020, 28, 13260–13268. 10.1364/OE.392052. 32403803

[ref19] Rivera P.; et al. Observation of long-lived interlayer excitons in monolayer MoSe2–WSe2 heterostructures. Nat. Commun. 2015, 6, 6242 10.1038/ncomms7242. 25708612

[ref20] Choi J.; et al. Twist Angle-Dependent Interlayer Exciton Lifetimes in van der Waals Heterostructures. Phys. Rev. Lett. 2021, 126, 047401 10.1103/PhysRevLett.126.047401. 33576642

[ref21] Robert C.; et al. Exciton radiative lifetime in transition metal dichalcogenide monolayers. Phys. Rev. B 2016, 93, 205423 10.1103/PhysRevB.93.205423. 27835018

[ref22] Jauregui L. A.; et al. Electrical control of interlayer exciton dynamics in atomically thin heterostructures. Science 2019, 366, 870–875. 10.1126/science.aaw4194. 31727834

[ref23] Kulig M.; et al. Exciton Diffusion and Halo Effects in Monolayer Semiconductors. Phys. Rev. Lett. 2018, 120, 207401 10.1103/PhysRevLett.120.207401. 29864294

[ref24] Ginsberg N.S.; Tisdale W.A. Spatially Resolved Photogenerated Exciton and Charge Transport in Emerging Semiconductors. Annu. Rev. Phys. Chem. 2020, 71, 1–30. 10.1146/annurev-physchem-052516-050703. 31756129

[ref25] Yuan L.; et al. Twist-angle-dependent interlayer exciton diffusion in WS2–WSe2 heterobilayers. Nat. Mater. 2020, 19, 617–623. 10.1038/s41563-020-0670-3. 32393806

[ref26] Li Z.; et al. Interlayer Exciton Transport in MoSe2/WSe2 Heterostructures. ACS Nano 2021, 15, 1539–1547. 10.1021/acsnano.0c08981. 33417424

[ref27] Koshino M.; Son Y.-W. Moiré phonons in twisted bilayer graphene. Phys. Rev. B 2019, 100, 075416 10.1103/PhysRevB.100.075416.

[ref28] Liu X.; Peng R.; Sun Z.; Liu J. Moiré Phonons in Magic-Angle Twisted Bilayer Graphene. Nano Lett. 2022, 22, 7791–7797. 10.1021/acs.nanolett.2c02010. 36170965 PMC9562463

[ref29] Uddin S. Z.; et al. Neutral Exciton Diffusion in Monolayer MoS2. ACS Nano 2020, 14, 13433–13440. 10.1021/acsnano.0c05305. 32909735

[ref30] Choi J.; et al. Moiré potential impedes interlayer exciton diffusion in van der Waals heterostructures. Sci. Adv. 2020, 6, eaba8866 10.1126/sciadv.aba8866. 32967823 PMC7531884

[ref31] O’Donnell K. P.; Chen X. Temperature dependence of semiconductor band gaps. Appl. Phys. Lett. 1991, 58, 2924–2926. 10.1063/1.104723.

[ref32] Huang J.; Hoang T. B.; Mikkelsen M. H. Probing the origin of excitonic states in monolayer WSe2. Sci. Rep 2016, 6, 22414 10.1038/srep22414. 26940069 PMC4778068

[ref33] Chernikov A.; et al. Electrical tuning of exciton binding energies in monolayer WS_2_. Phys. Rev. Lett. 2015, 115, 126802 10.1103/PhysRevLett.115.126802. 26431003

[ref34] Latini S.; Olsen T.; Thygesen K. S. Excitons in van der Waals heterostructures: The important role of dielectric screening. Phys. Rev. B 2015, 92, 245123 10.1103/PhysRevB.92.245123.

[ref35] Kośmider K.; González J. W.; Fernández-Rossier J. Large spin splitting in the conduction band of transition metal dichalcogenide monolayers. Phys. Rev. B 2013, 88, 245436 10.1103/PhysRevB.88.245436.

[ref36] Chatterjee S.; et al. Harmonic to anharmonic tuning of the moiré potential leading to unconventional Stark effect and giant dipolar repulsion in WS2/WSe2 heterobilayer. Nat. Commun. 2023, 14, 4679 10.1038/s41467-023-40329-3. 37542024 PMC10403536

[ref37] Wu F.; Lovorn T.; MacDonald A. H. Theory of optical absorption by interlayer excitons in transition metal dichalcogenide heterobilayers. Phys. Rev. B 2018, 97, 035306 10.1103/PhysRevB.97.035306.

[ref38] Jin C.; et al. Identification of spin, valley and moiré quasi-angular momentum of interlayer excitons. Nat. Phys. 2019, 15, 1140–1144. 10.1038/s41567-019-0631-4.

[ref39] Yu H.; Liu G.-B.; Yao W. Brightened spin-triplet interlayer excitons and optical selection rules in van der Waals heterobilayers. 2D Mater. 2018, 5, 035021 10.1088/2053-1583/aac065.

[ref40] Barré E.; et al. Optical absorption of interlayer excitons in transition-metal dichalcogenide heterostructures. Science 2022, 376 (6591), 406–410. 10.1126/science.abm8511. 35446643

[ref41] Nayak P. K.; et al. Probing Evolution of Twist-Angle-Dependent Interlayer Excitons in MoSe2/WSe2 van der Waals Heterostructures. ACS Nano 2017, 11 (4), 4041–4050. 10.1021/acsnano.7b00640. 28363013

[ref42] Penzo E.; et al. Long-Range Exciton Diffusion in Two-Dimensional Assemblies of Cesium Lead Bromide Perovskite Nanocrystals. ACS Nano 2020, 14, 6999–7007. 10.1021/acsnano.0c01536. 32459460

[ref43] Cadiz F.; et al. Exciton diffusion in WSe2 monolayers embedded in a van der Waals heterostructure. Appl. Phys. Lett. 2018, 112, 152106 10.1063/1.5026478.

[ref44] Wagner K.; et al. Nonclassical Exciton Diffusion in Monolayer WSe2. Phys. Rev. Lett. 2021, 127, 076801 10.1103/PhysRevLett.127.076801. 34459627

[ref45] Akselrod G. M.; et al. Visualization of exciton transport in ordered and disordered molecular solids. Nat. Commun. 2014, 5, 3646 10.1038/ncomms4646. 24736470

[ref46] Glazov M. M. Quantum Interference Effect on Exciton Transport in Monolayer Semiconductors. Phys. Rev. Lett. 2020, 124, 166802 10.1103/PhysRevLett.124.166802. 32383933

[ref47] Glazov M. M.; Iakovlev Z. A.; Refaely-Abramson S. Phonon-induced exciton weak localization in two-dimensional semiconductors. Appl. Phys. Lett. 2022, 121, 192106 10.1063/5.0122633.

[ref48] Wagner K.; et al. Diffusion of exciton in a two-dimensional Fermi Sea of charges. Nano Lett. 2023, 23, 4708–4715. 10.1021/acs.nanolett.2c03796. 37220259

[ref49] Guo H.; Zhang X.; Lu G. Moiré excitons in defective van der Waals heterostructrures. Proc. Natl. Acad. Sci. U. S. A. 2022, 118 (32), e2105468118 10.1073/pnas.2105468118. PMC8364154 34341106

[ref50] You Y.; et al. Observation of biexcitons in monolayer WSe_2_. Nat. Phys. 2015, 11, 477–481. 10.1038/nphys3324.

[ref51] Courtade E.; et al. Charged excitons in monolayer WSe_2_: Experiments and theory. Phys. Rev. B 2017, 96, 085302 10.1103/PhysRevB.96.085302.

[ref52] Maity I.; Mostofi A. A.; Lischner J. Electrons Surf Phason Waves in Moiré Bilayers. Nano Lett. 2023, 23 (11), 4870–4875. 10.1021/acs.nanolett.3c00490. 37235740 PMC10273461

[ref53] Büch H.; et al. Superlubricity of epitaxial monolayer WS2 on graphene. Nano Res. 2018, 11, 5946–5956. 10.1007/s12274-018-2108-7.

[ref54] Yuan J.; Yang R.; Zhang G. Structural superlubricity in 2D van der Waals heterojunctions. Nanotechnology 2022, 33, 102002 10.1088/1361-6528/ac1197. 34229304

[ref55] Wang L.; et al. One-Dimensional Electrical Contact to a Two-Dimensional Material. Science 2013, 342, 614–617. 10.1126/science.1244358. 24179223

[ref56] Yuan L.; et al. Twist-angle-dependent interlayer exciton diffusion in WS2–WSe2 heterobilayers. Nat. Mater. 2020, 19, 617–623. 10.1038/s41563-020-0670-3. 32393806

[ref57] Naik S.; Naik M. H.; Maity I.; Jain M. Twister: Construction and structural relaxation of commensurate moiré superlattices. Comput. Phys. Commun. 2022, 271, 108184 10.1016/j.cpc.2021.108184.

[ref58] Jiang J.-W.; Zhou Y.-P.; Jiang J.-W.; Zhou Y.-P.; Handbook of Stillinger-Weber Potential Parameters for Two-Dimensional Atomic Crystals; IntechOpen, 2017. ISBN: 978–953–51–3696–5.

[ref59] Thompson A. P.; et al. LAMMPS - a flexible simulation tool for particle-based materials modeling at the atomic, meso, and continuum scales. Comput. Phys. Commun. 2022, 271, 108171 10.1016/j.cpc.2021.108171.

[ref60] Togo A.; Tanaka I. First principles phonon calculations in materials science. Scripta Materialia 2015, 108, 1–5. 10.1016/j.scriptamat.2015.07.021.

[ref61] Soler J. M.; et al. The SIESTA method for ab initio order-N materials simulation. J. Phys.: Condens. Matter 2002, 14, 2745 10.1088/0953-8984/14/11/302.

[ref62] Fernández-Seivane L.; Oliveira M. A.; Sanvito S.; Ferrer J. On-site approximation for spin–orbit coupling in linear combination of atomic orbitals density functional methods. J. Phys.: Condens. Matter 2006, 18, 7999 10.1088/0953-8984/18/34/012.

[ref63] Perdew J. P.; Zunger A. Self-interaction correction to density-functional approximations for many-electron systems. Phys. Rev. B 1981, 23, 5048–5079. 10.1103/PhysRevB.23.5048.

