# Supplementary material for: Anomalous Interlayer Exciton Diffusion in WS2/WSe2 Moiré Heterostructure
Source: ACS Nano. 2024 Jul 1;18(28):18202–10. doi: 10.1021/acsnano.4c00015 (PMC11256890; doi:10.1021/acsnano.4c00015)
Supplement: Supplementary file 1 — nn4c00015_si_001.pdf [file nn4c00015_si_001.pdf]

Supporting information: Anomalous interlayer exciton diffusion in WS_2/WSe_2 moiré heterostructure

*Antonio Rossi[1,2,3] *‡, Jonas Zipfel[1] ‡ Indrajit Maity[4] ‡, Monica Lorenzon[1], Medha Dandu[1], Elyse Barré[1], Luca Francaviglia[1], Emma C. Regan[5], Zuocheng Zhang[5], Jacob H. Nie[5,6], Edward Barnard[1], Kenji Watanabe[7], Takashi Taniguchi[8], Eli Rotenberg[2], Feng Wang[5], Johannes Lischner[4], Archana Raja[1] * and Alexander Weber-Bargioni[1] **

1-The Molecular Foundry, Lawrence Berkeley National Laboratory, Berkeley, 94720, USA.

2-Advanced Light Source, Lawrence Berkeley National Laboratory, Berkeley, 94720, USA.

3-Center for Nanotechnology Innovation @ NEST, Instituto Italiano di Tecnologia, Pisa, 56127, Italy

4-Imperial College London, South Kensington Campus, London, SW7 2AZ, United Kingdom.

5-Department of Physics, University of California at Berkeley, Berkeley, CA, 94720, USA.

6-Department of Physics, University of California at Santa Barbara, Santa Barbara, CA, 93106, USA

7-Research Center for Functional Materials, National Institute for Materials Science, 1-1 Namiki, Tsukuba, 305-0047, Japan

8-International Center for Materials Nanoarchitectonics, National Institute for Materials Science, 1-1 Namiki, Tsukuba, 305-0047, Japan

‡-These authors contributed equally

*Corresponding authors: antonio.rossi@iit.it, araja@lbl.gov, awb@lbl.gov

Figure S1: Sample Characterization

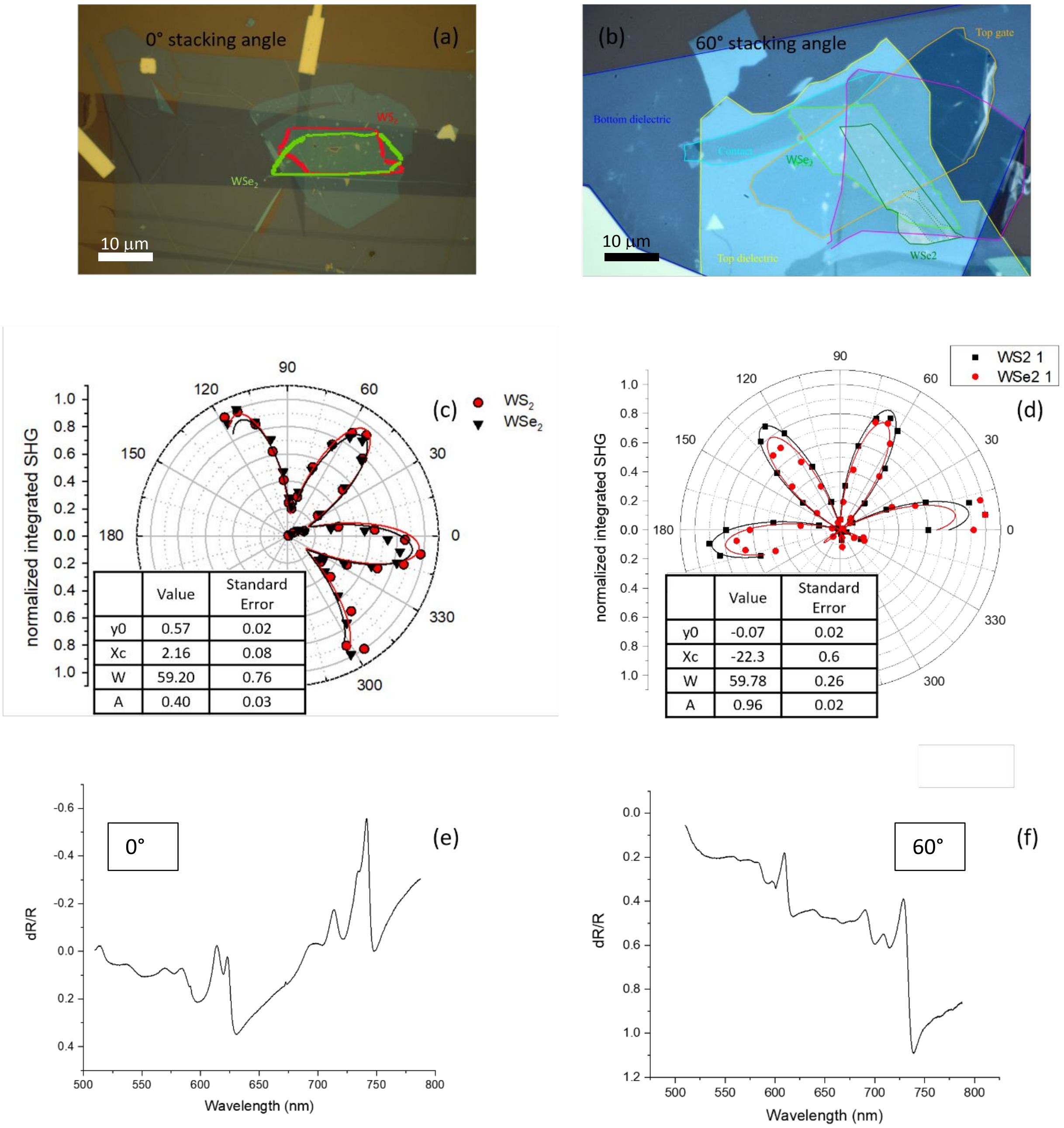

	Value	Standard Error
y0	0.57	0.02
Xc	2.16	0.08
W	59.20	0.76
A	0.40	0.03

	Value	Standard Error
y0	-0.07	0.02
Xc	-22.3	0.6
W	59.78	0.26
A	0.96	0.02

Figure S1. Optical image of the samples used for this work. (a) and (b) are images of the 0° and 60° stacking angle samples respectively. (c-d) Second Harmonic Generation (SHG) analysis confirming the relative orientation of WS2 and WSe2 monolayers to be 0° and 60 respectively. (e-f) Adsorption spectra for the two samples. The sample in panel (a) is the same sample used in Ref.1.

Figure S2: PL spectrum multipeak fitting

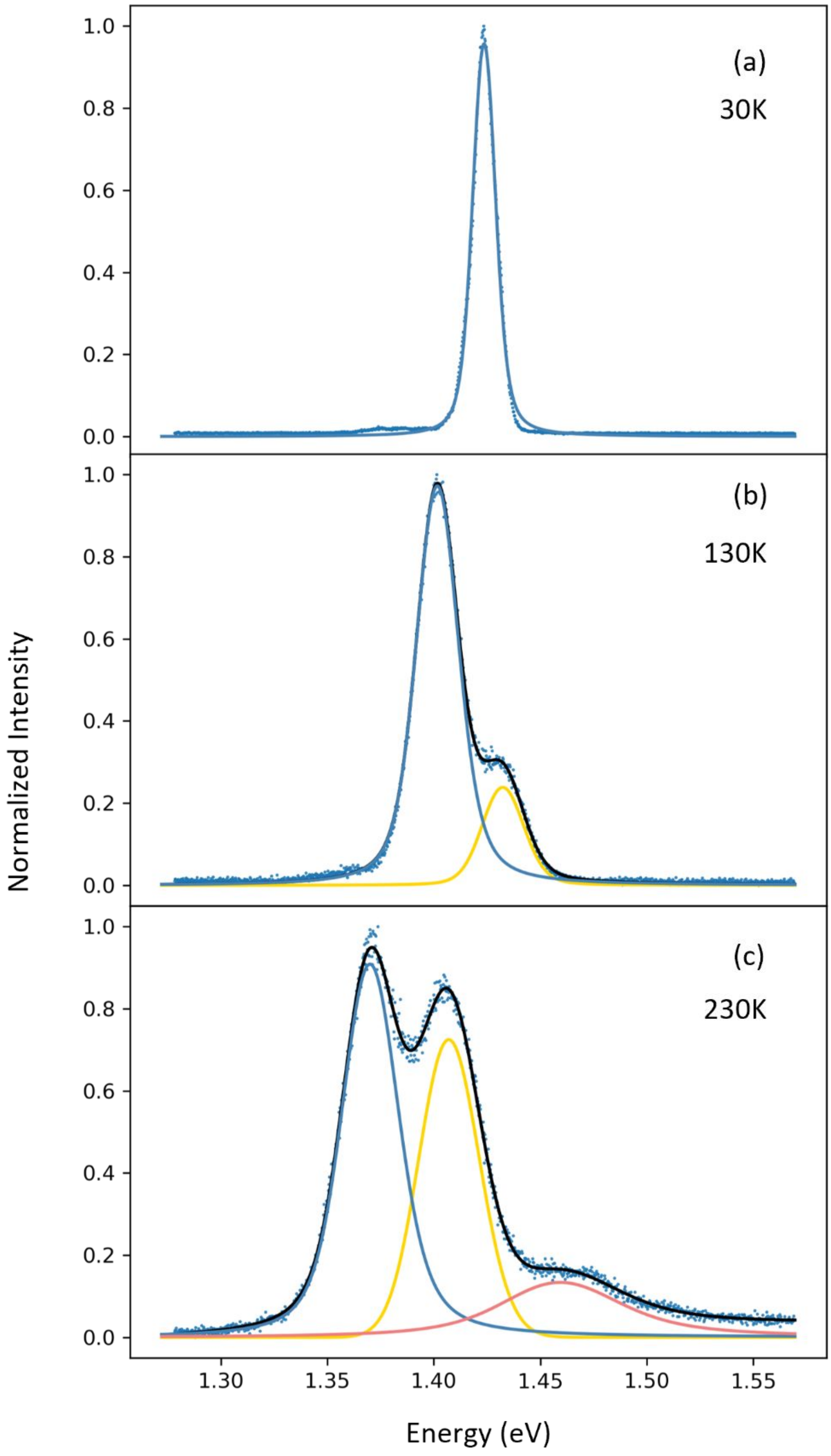

Figure S3: Diffusion Measurements

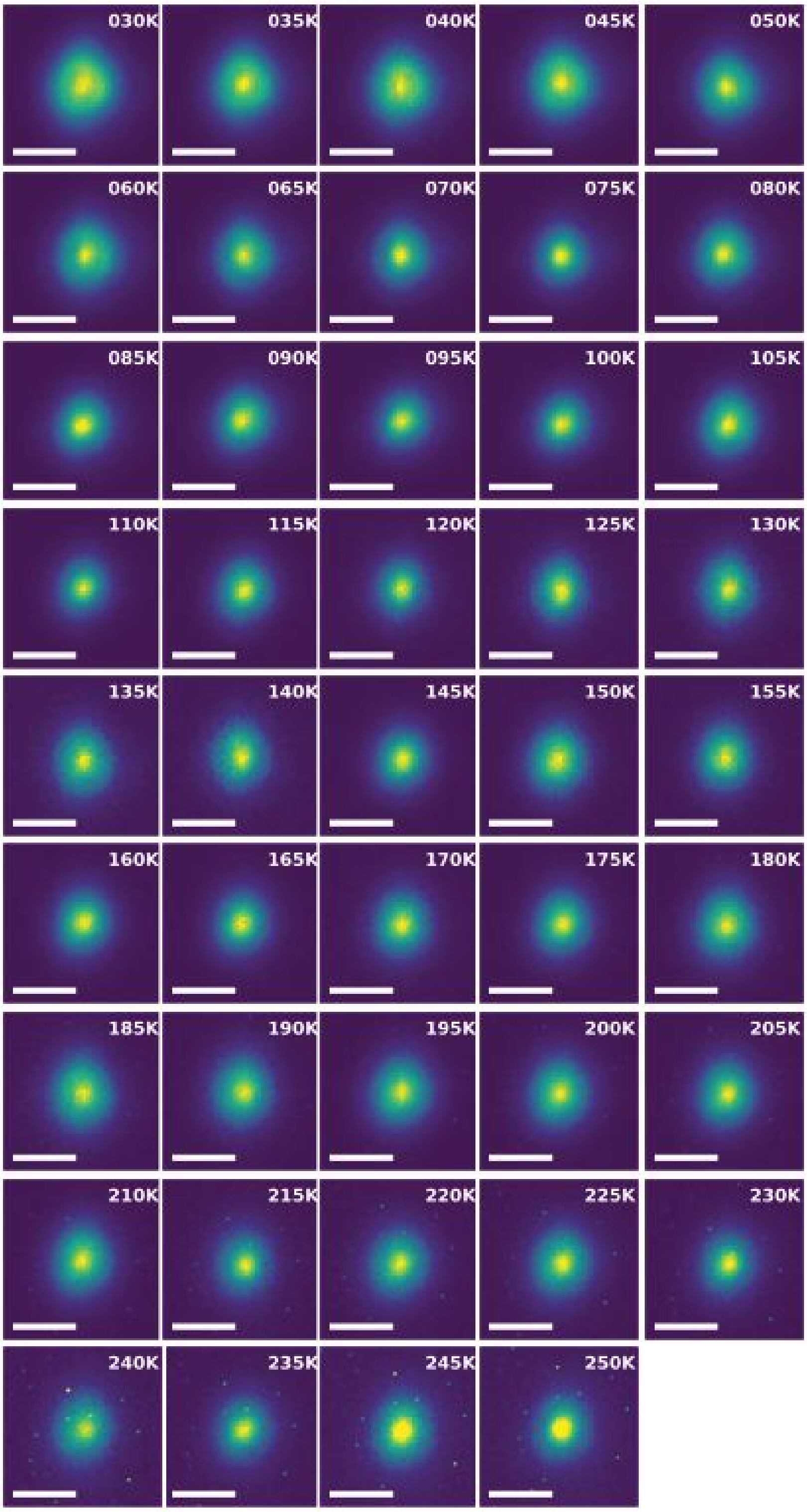

Figure S4: Diffusion Measurements

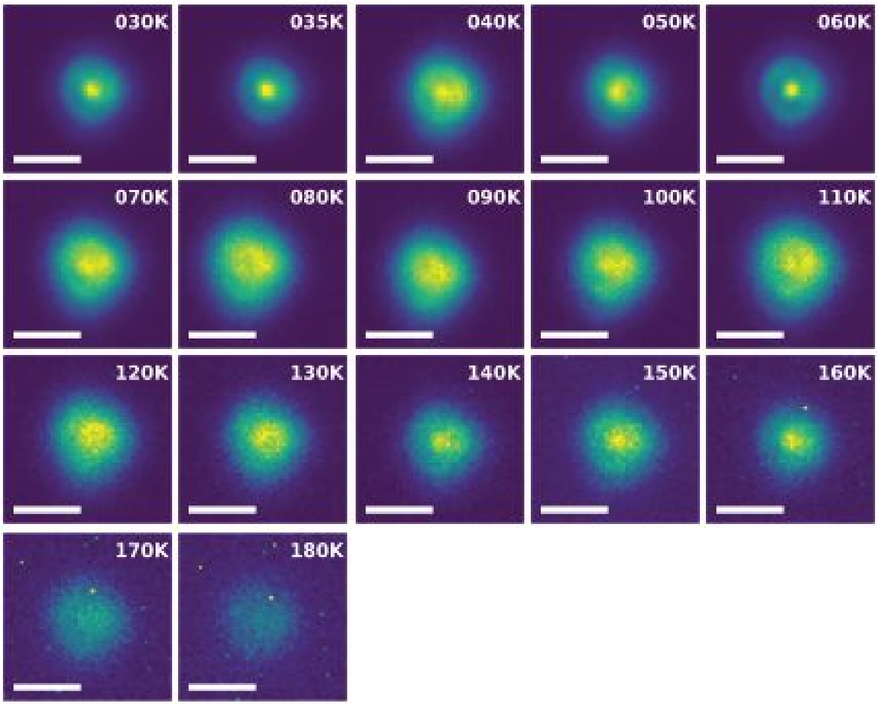

Figure S4. Spatially resolved PL emission from 0° stacking angle sample. Each image is taken at different temperatures, ranging from 30 K to 180 K. The scale bar is 2 μm.

Figure S5: Exciton Diffusion single layer

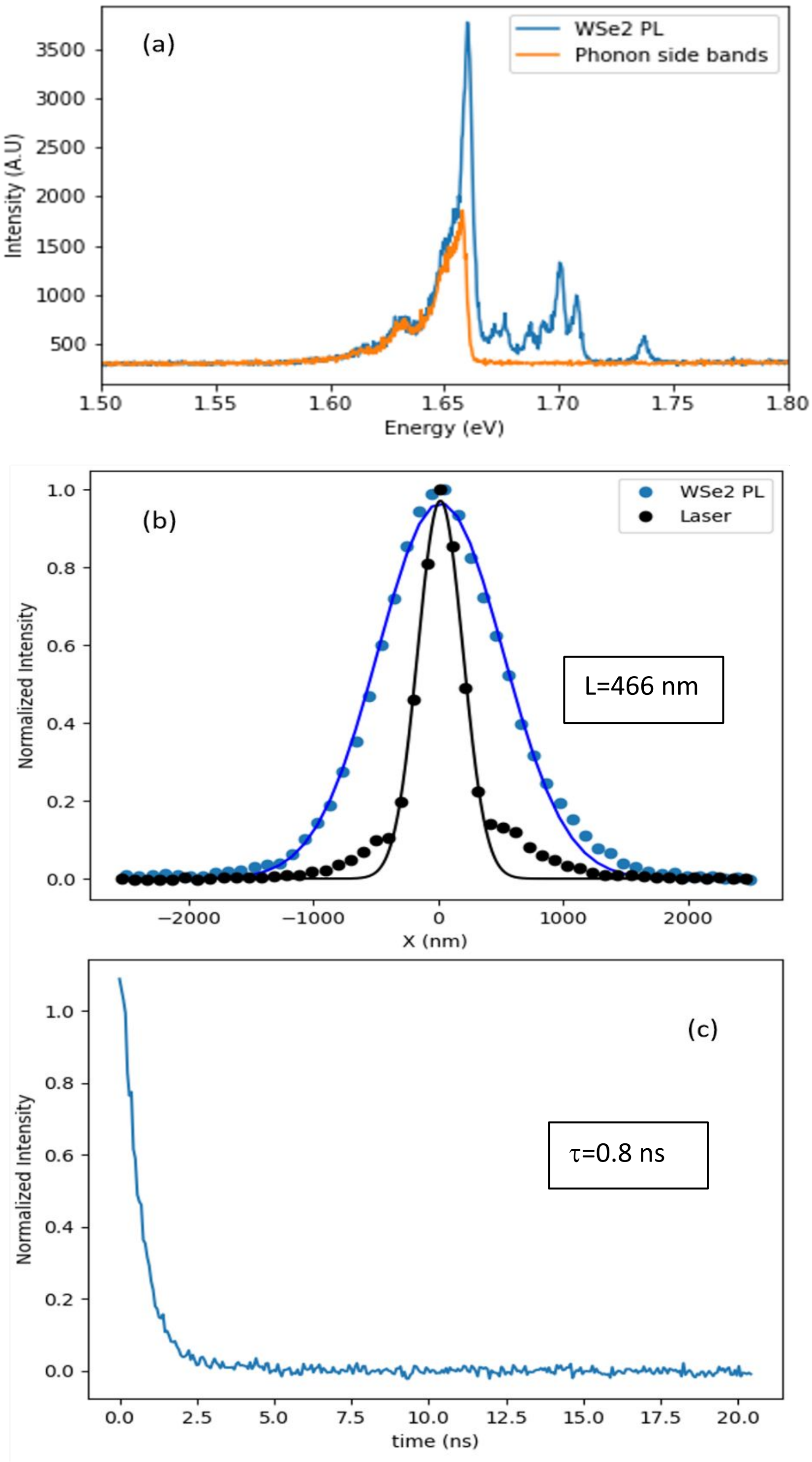

Figure S5. To estimate the exciton diffusivity at cryogenic temperature in monolayer we measure the dark exciton dynamic through their spectrally well separated phonon side-bands (PSB[12]. (a)PL spectra of WSe_2 (blue) and PSB only (orange). Spatially(b) and (c) time resolved PL from PSB of isolated WSe_2 crystal The extracted diffusion length and lifetime are reported in the insets. The calculated Diffusivity is D = 1.35 cm^2/s

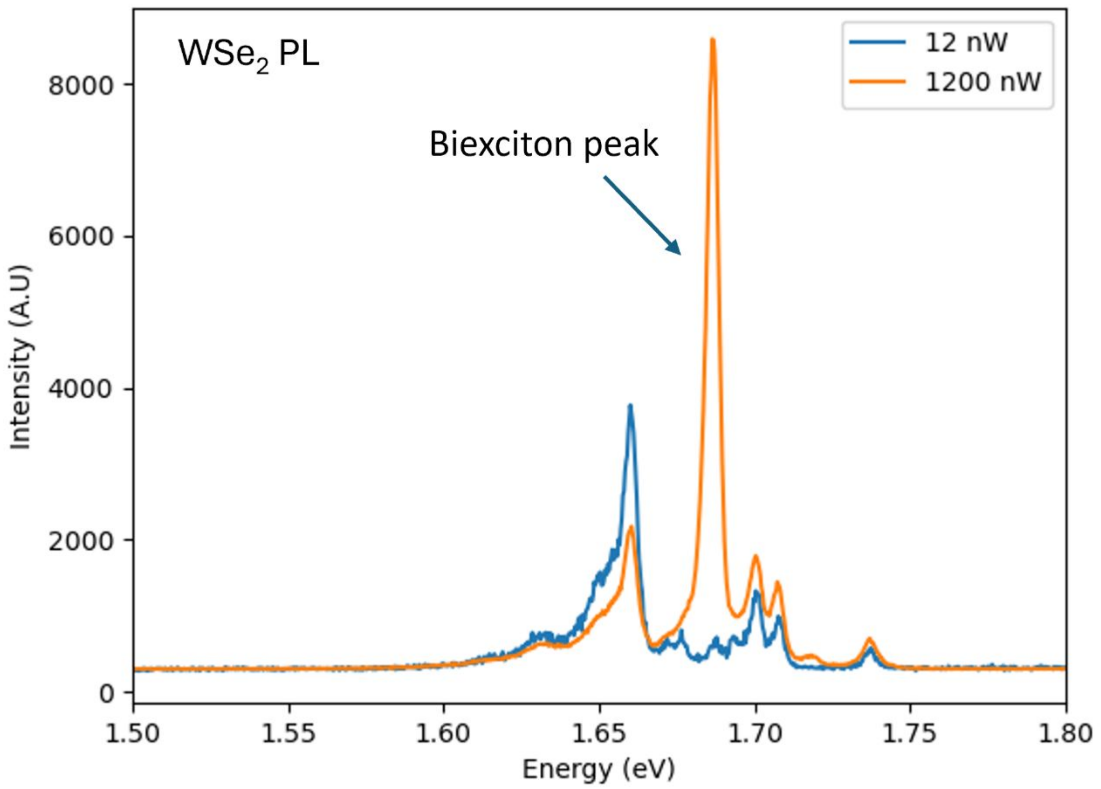

Figure S6 PL spectra of a WSe_2 monolayer measured at two different illumination intensities. Notably, a biexciton peak[3] emerges exclusively under high-power illumination conditions, whereas at the lower power settings utilized for our interlayer exciton (IX) experiments, this feature is absent.

Figures S7-S8-S9: Additional details on DFT Calculations

A. Moiré potential using unit-cell calculations

The unit-cell lattice-constants of the WS_2 and WSe_2 are 3.18 and 3.32 Å, respectively. The lattice mismatch (about 4%) gives rise to a moiré when WS_2 and WSe_2 are stacked together even without a twist angle. We extract the moiré potential using the approach discussed in the main text. We also strain both the layer (isotropic tensile strain for WS_2 and compressive strain for WSe_2) and set the unit-cell lattice to 3.25 Å. We find that the qualitative moiré potential differences between 0◦ and 60◦ stacking match the experiments (see Fig. S7). However, these unit-cell calculations that do not include atomic reconstructions hugely underestimate the moiré potential for exciton hopping (which were shown in the main text). All the calculations are performed with the Quantum ESPRESSO package[4].

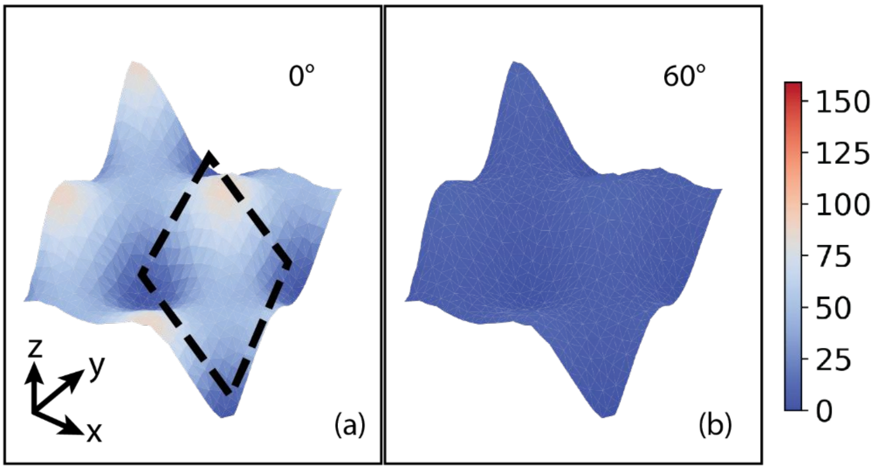

B. Moiré potential extraction from twisted bilayer

We develop a simple scheme to compute the moiré potential using large-scale DFT calculations by taking into account the full atomic reconstructions. In this approach, we explicitly consider the localization of the electronic wave functions and extract the local band gap for different high symmetry stacking regions. To illustrate this, we plot the electronic wave function $|\psi_\Gamma|^2$ for several bands near the band gap (see Fig. S8). The valence bands are marked as V1, V2,... . V1 corresponds to Valence Band Maximum (VBM). Similarly, the conduction bands are marked as C1, C2,... . C1 corresponds to Conduction Band Minimum (CBM). The local band gap at the red diamond stacking is 0.775 eV and obtained from V2 and C1 bands. The local band gap at the orange diamond stacking is 0.843 eV and obtained from V1 and C4 bands. The local band gap at the green diamond stacking is 0.963 eV and obtained from V3 and C4 bands. Similarly, we obtain the local band-gaps for 0◦ twisted WS_2/WSe_2 heterobilayer.

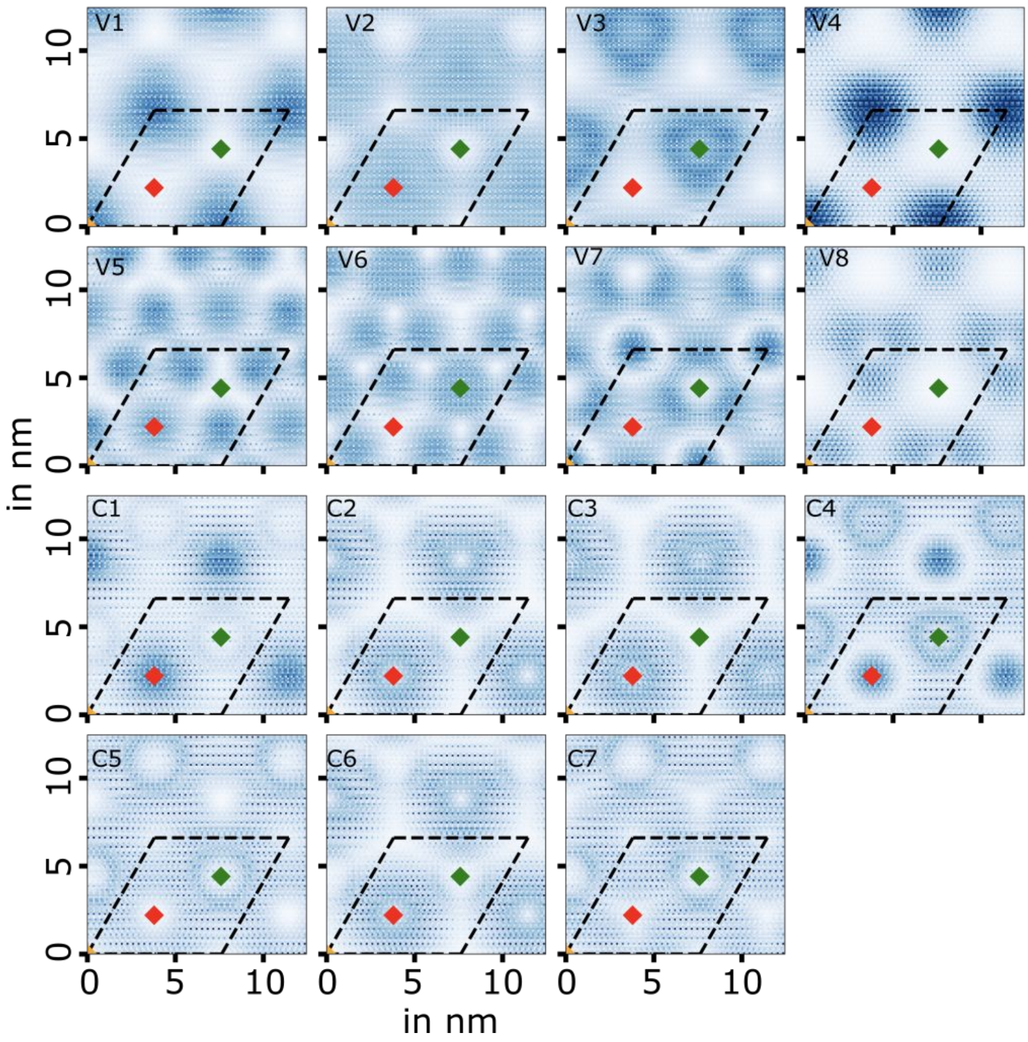
V1
V2
V3
V4
V5
V6
V7
V8
C1
C2
C3
C4
C5
C6
C7
in nm
in nm
0
5
10

C. Moiré movements at different times

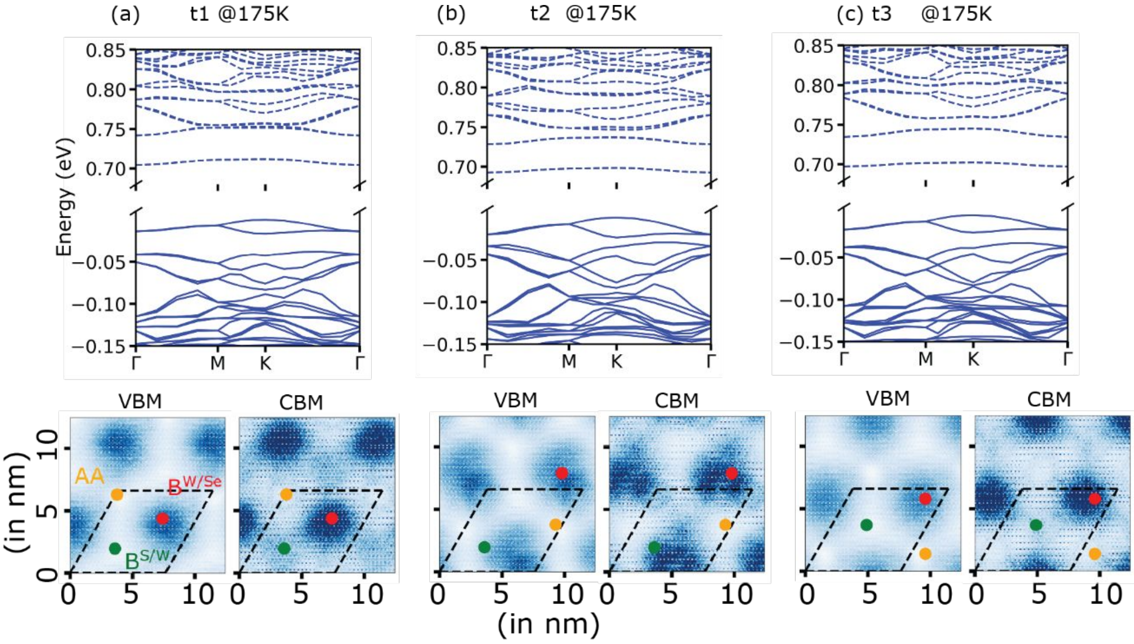

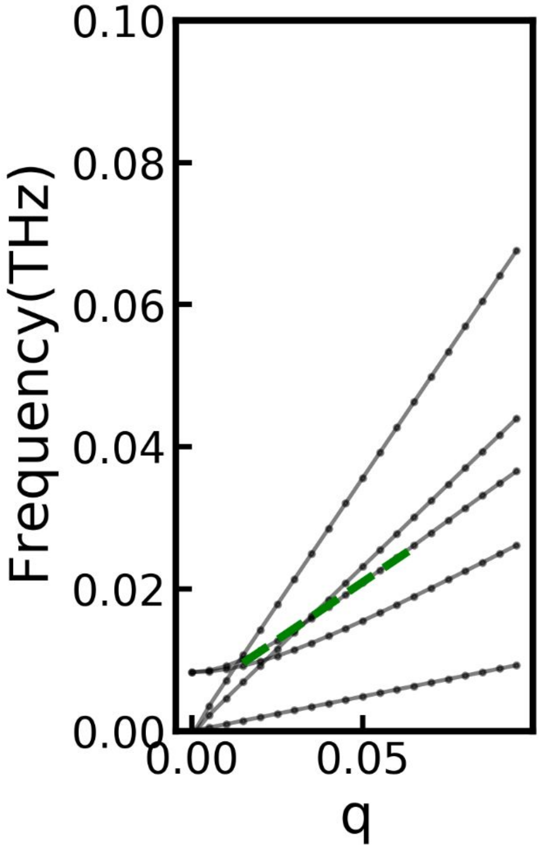

Figure S10 Phason dispersion fitted with a linear function (green dashed line) to extract the phason group velocity near the Γ point. The parabolic dispersion makes the fitting less accurate approaching Γ.

References

1. Jin, C. *et al.* Identification of spin, valley and moiré quasi-angular momentum of interlayer excitons. *Nat. Phys.* **15**, 1140–1144 (2019).
2. Rosati, R. *et al.* Temporal Evolution of Low-Temperature Phonon Sidebands in Transition Metal Dichalcogenides. *ACS Photonics* **7**, 2756–2764 (2020).
3. You, Y. et al., Observation of biexcitons in monolayer WSe2, *Nat. Phys 11, 477-481 (2015)*
4. Giannozzi, P. *et al.* QUANTUM ESPRESSO: a modular and open-source software project for quantum simulations of materials. *J. Phys.: Condens. Matter* **21**, 395502 (2009).
